# Psychometric properties of Arabic translated temperament instrument

**DOI:** 10.1016/j.amsu.2021.102341

**Published:** 2021-04-21

**Authors:** Sawsan Abuhammad, Manar Al-Azzam, Nasr Alrabadi, Kimberly Howard, Rana AbuFarha

**Affiliations:** a Jordan University of Science and Technology/Faculty of Nursing, PO Box 3030, Irbid, 22110, Jordan; b Al-albayt University, Mafrq, Jordan; c College of Medicine, Jordan University of Science and Technology, Irbid, 2211, Jordan; d Information Science and Research Specialist, Master of Library and Information Science (MLIS), Jordan; e Pharma –Applied Science Private University, Amman, Jordan

**Keywords:** Infant temperament, Secure attachment, Translation, Jordan

## Abstract

**Background:**

Investigating temperament has been a multi-subject exercise that attempts to determine the contribution of temperament to additional measurable phenomena like behavior. The existing research not only evaluates temperament as a variable with the ability to influence additional characteristics but has included interventions that can result in adapted outcomes. Develop an Arabic translation of the Infant Characteristics Questionnaire (ICQ).

**Purpose:**

Examine the psychometric properties of the translation and establish modified criteria for evaluating the questionnaire.

**Methods:**

Cross sectional design was used. Translation of the ICQ was achieved through translation/back translation. The population that used the ICQ translation on convenience sample of 40 Arabic-speaking women with children between the ages of 3–12‐months old; this same group also completed a demographic survey.

**Results:**

The internal consistency of the translated instrument was almost equivalent to or higher than the reported from the United States (US) review. The alpha coefficients calculated from subscales varied between .47 and .87.

**Conclusion:**

This research study described the translation of the ICQ into the Arabic language for use in the research and clinical setting. The ICQ is a useful tool for evaluating infant difficulty by describing and summarizing parents’ ratings.

## Highlights

• In nursing for infants, the parent-child, is very significant and is often understood as bilateral, correlative and interdependent.
• Infant temperament is described as regularities in responding to different situations, and reflect a infant's behavioral characteristics.
• The information gathered from infant-temperament questionnaires can help health care professionals inform parents of interventions for improving infant temperament.

## 1 Introduction

Investigating phenomenon can be aided by the translation of research instrumentation from a source language to a target language. However, researchers must consider semantic, conceptual, and normative likeness or the instrument, and the data it was used to gather, could be consider useless [1,2]. As research using human sample populations is growing increasingly widespread, investigators must answer the question how a survey instrument can be adjusted for use in multiple countries when it was prepared in a different language [1]. One of these areas are infant temperament that required validated instrument that could use in different languages and countries. Infant temperament is described as regularities in responding to different situations, and as a classification of an infant's behavioral characteristics [[3], [4], [5]]. This phenomenon has frequently been analyzed through the parent-infant relationship, via parent's completion of self-report questionnaires related to their infant's behavior. Many studies examine the relationship between the infant's temperament and the parental or caregiver relationship, for example, on parental responsiveness, and the influence of temperament on the child's externalizing behavior [6]. This literature helps with understanding the role of the correlative parent-infant relationship, the impact of parental practices, and the investigation of temperament as a variable ultimately contributing to the child's working model, or the way they seem themselves, and behave in the world [6].

The information gathered from infant-temperament questionnaires can help health care professionals inform parents of interventions for improving infant temperament, as well as the bilateral parent-child relationship [3]. Since it is known that the temperament data can inform intervention strategies regard the child's emotional status, the availability of a measurement mechanism is a very meaningful tool for health care professionals and the caregivers they support [7]. Ultimately, mediation is also very important to the infants themselves whose lives are significantly impacted by their caregivers' quality, as indicators show inconsistent or withdrawn caregiver behavior can lead to higher levels of emotional or behavioral problems in children that can persist later life [7,8]. Building on the work of Thomas and Chess, the Infant Characteristic Questionnaire (ICQ) designed by Bates [9] is a reliable, cross-validated, internally consistent, and factor-analytic tool, for measuring the level of infant difficultness by querying parent's and independent researchers' responses [9]. There are additional tools for infant temperament measurement, however, this paper and research study was based on the ICQ method. This research study evaluates the psychometric properties of the Arabic language translation of the Infant Characteristics Questionnaire (ICQ) [8]. The investigators examined: 1) best practices for translating from a source language into the target language, 2) tested the reliability and validity of the translation by comparing the relevant statistical retest data, and 3) examined both consistencies and inconsistencies between the primary questionnaire and the translation.

## 2 Methods

### 2.1 Participant

The translated Arabic-language ICQ was distributed to sample of 40 Arabic-speaking mothers of two-to twelve-month-old healthy full-term babies.

### 2.2 Recruitment and procedure

The research team used the snowball method to recruit participants, meaning participants invited people known to them to participate in the study [10]. The participant packet included an ICQ, a demographic questionnaire, a pre-stamped return envelope and explanation of the study. The questionnaires were returned via traditional mail; (50) were distributed, (43) were returned, (17) were excluded due to missing age data, and [3] were excluded due to infant age not meeting, or exceeding the restriction for the study parameters.

### 2.3 Instrument

The *Infant Characteristic Questionnaire (ICQ*) has 24 items, with responses ranging from Ref. [1] optimal temperament to Ref. [7] difficult temperament on a seven-point Likert scale; this questionnaire was used to assess the maternal, or parental, view of the infant's temperament as father's were also queried. The items in the survey were based on existing literature including the types of temperament, changeability and soothability criteria, and fussiness and sociability consideration as indicated by previous studies; the survey also showed that the parent's view of infant difficultness can be influenced by parental traits or characteristics [9]. The questionnaire was self-described by the researchers that developed it as short, with investigators mentioning the length as a potential weakness, but also a strength in that completion rates were high [9]. Researchers also indicated that despite being short, it rendered stable interpretable factor structure, showed sufficient test-retest stability and was psychometrically valid [9].

### 2.4 Translation

When translating a research questionnaire from a source language into a target language various issues can arise [11]. The ability to achieve semantic equivalence, or the same meaning, is a precursor to the achievement of reliability, and in short, a successful translation [1]. While the use of questionnaires for data gathering could currently be described as a normality, multilingual survey uses and adaptation are less of a commonality, making the description of the methodologies used, the evaluation of the validity and internal consistency of the translated instrument relevant and potentially instructional. Investigators purport that documenting difficulties that arise and the kinds of errors that can result is noteworthy as it can provide clarity into the process of translation and cross-cultural research [11]. There can be very significant problems in translation in such areas as vocabulary, idiomatic, grammatical-syntatical, experiential, and conceptual equivalence [11]. For example, it can be impossible finding words or expressions that mean the same thing in the target language, and direct translation might not have the intended meaning; a single direct translation might not reveal any problems in translation [11]. In addition, at times the preservation of the idea is more important than literal linguistic equivalence [11]. Translation-back translation which can be described as having singular, or groups, of bilingual translators complete the first translation from the mother language to the expected language, and then using the additional translator(s) to translate from the expected language back into the mother language, is useful in finding inconsistencies, as the review process continues until superior consensus is reached [1]. This is the translation method used in this research study and which will be further described in the following sections.

The translation back-translation method was used to make an instrument that was equivalent in meaning to the source language instrument. The investigators in this study used the World Health Organization (WHO) method which emphasizes cross-cultural and conceptual translation and not verbatim word-based equivalence. The WHO method consist of 1) Forward translation of the version, 2) Expert panel 3) Pre-testing of the version and cognitive interviewing of the participant, and 4) Final version [12]. Step 1: The first translator, translator A, was a bilingual, English-Arabic healthcare professional whose mother language was Arabic. This translator was tasked with producing the first translation also known as the forward translation and was directed to aim for conceptual rather than linguistic equivalence and use natural rather than pedantic language. The result from this translation was version 1. The second translator was a subject matter expert, who was also bilingual English-Arabic, whose mother tongue was Arabic. This reviewer, translator B, reviewed the first translation for inadequate expressions and concepts and recommended substitutions. Updating was completed using the feedback resulting in version 2. Step 2: The research team, also served the function of the expert panel. They reviewed the translation and recommended further changes resulting in version 3. Step 3: The third translator, translator c, was a bilingual native speaker of English that was not a subject matter expert. This translator back-translated the forward translation and was given the same directives regarding concept-based rather than literal translation. The goal at this step was to identify problematic words or phrases that when back-translated from the original were not equivalent in the meaning to the original ICQ. The research team and primary investigators again reviewed the problematic language, identified, and used consensus to inform the editing process resulting in version 4. Step 4: The fourth step consisted of pretesting and cognitive interviewing of a pretest population of 10 Arabic parents with infants less than seven months after childbirth. The information of the study was explained to the pretest population; they were also asked to a) Restate the question using their words, b) Explain what they thought was being asked by each item, c) Explain what they understood of keywords and phrases, d) Describe what thinking process they used to select their answer. After reviewing the problems identified in the pre-test such as comprehension and semantic problems, the research team convened to consider further changes and the final version of the translation, version 5 was finalized. This paper was checked to meet all the criteria of STROSS criteria [10].

### 2.5 Ethical concerns

An agreement to conduct the study was obtained from Jordan University of Science and Technology. The IRB number is (2017232). The research participants received written and verbal details regarding the study including the aim, directives on how to participate or withdraw. Prospective participants were also ensured that they would be unidentifiable, and that study data would be kept classified [10]. It is also registered with Research Registry with number research registry 6670 https://www.researchregistry.com/browse-the-registry#user-researchregistry/registerresearchdetails/60548e4ae6df0f001c9fadb8.

### 2.6 Statistical analysis

The researcher used SPSS (25 version) to analyze the data. The researchers used cronbach's alpha coefficient (α) for every subscale and for the total child temperament questionnaire to assess the internal consistency of the questionnaire, values ≥ 0.60 were considered satisfactory. Exploratory principal component analysis (PCA) using varimax rotation methods was used of the Arabic translations of child temperament tool to evaluate construct validity and to assess if measured just one component. Eigen values and a screen plot were examined to determine the number of sub items. Eigen values were used to describe the degree of variance of each construct in the study. Every item got value of 1 or more will be considered in the questionnaire as component (Kaiser's criterion; [13]).

## 3 Results

### 3.1 Descriptive

The demographic data for the research study participants is shown in Table 1, and further described herein. The mother's age ranged from 18 to 40 years, 47.5% *(n* = 19) mothers did not have the equivalent of a high school level education, 77.5%, (*n* = 31) mothers were without employment; the majority of mother's lived with extended family 77.5% (*n* = 31). The infant mean age was reported at 8 months, while the infant age range was three to twelve months; about 55% (*n* = 22) were female, while 45% (*n* = 18) were male. Nearly 26.2% (*n* = 18) received assistance providing care for infants, with 65% (*n* = 70) mother's receiving support from grandparents. Most of the mother's reported previous experience with infants at 65% (n = 26); the greater part also reported a good relationship between wife and husband at 57.5% (*n* = 23), compared with just 2.5% (*n* = 1) reporting a negative relationship between wife and husband.

**Table 1:** Characteristics of the study participants (N = 40).

Parameters	n (%)	Mean (SD)
**Mother age (years)**		29.2 (6.1)
**Educational level**		
•Primary	4 (10.0)	
•Secondary	17 (42.5)	
Diploma	19 (47.5)	
•Bachelor or more	0 (0)	
**Employment status**		
•Employed	9 (22.5)	
•Not employed	31 (77.5)	
**Assistance in infant care**		
•Yes	18 (46.2)	
•No	21 (53.8)	
**Experience in infant care**		
•Yes	26 (65.0)	
•No	14 (35.0)	
**Living in Independent house**		
•Yes	31 (77.5)	
•No	9 (22.5)	
**Perception towards marital relationship after childbirth** •Very good •Good •Fair •Bad	23 (57.5) 11 (27.5) 5 (12.5) 1 (2.5)	
**Family income level (Jordanian dinar)**		384.0 (160.9)
**Infant age (months)**		6.3 (3.6)
**Infant gender**		
•Male	18 (45.0)	
•Female	22 (55.0)	

a One Jordanian dinar is equal to 1.4 US dollars.

### 3.2 Face validity

Face validity is described as a subjective assessment of whether the research measures the phenomena it intends to measure; this measurement is completed to make sure the research assessment is valid at face value, ie. it is a type of test to determine whether the research instrumentation accurately reflects what investigators claim to be measuring. In this research study, face validity was measured by having non-experts evaluate whether the research instrument prompts represented the reported aims of the research study. After the instrument was translated, the research instrument was given to a group of four randomly selected mothers and two students from grades six and nine, to test the intelligibility of the research questions. These subjects were provided with information on the reason and goals of the study and were then asked to report if the questions were fitting, well-defined, easily comprehended, and appropriate based on the rationale for the study. The face validity test group participants reported the instrument to be easily understood and applicable. Due to this review, the instrument was found to have high face validity.

### 3.3 Content validity

Content validity is a type of assessment that examines how well a test measures the elements of a given construct, ie. This kind of assessment could be said to consider the field of study and whether the instrument is valid according to what it should measure given the constructs and definitions in that field, rather than just examining whether the questions look appropriate at general, non-subject-matter expert face value. The content validity for this research study was examined by the investigators to make sure that the instrument was complete and thorough enough to be used to answer the research area of investigation and study aims. After an initial review by the study investigators, the study questionnaire was given to three independent professors of nursing [2] and nutrition [1]. The expert group was asked to do an item-level rating of each prompt on the questionnaire using the Content Validity Index (CVI) scale ranging from: highly relevant, quite relevant, somewhat relevant, and not relevant. For the study investigators to include the item in the final version of the questionnaire it had to receive one of the two highest ratings: highly relevant or quite relevant. The following four items were excluded: How many times you baby get fussy? What the extent of crying and fussy? what extent for being wanting to hold? How many times smile or be happy? Moreover, the experts suggested change the likert scale to fiver instead of seven to decrease the error. They also suggested using imperative sentence to be more understandable for Arabic Families.

Lynn [14] suggests that when an instrument is evaluated by less than five subject-matter authorities, the threshold for item inclusion should be three quite relevant or highly relevant scores from at least three of the specialists, and anything less than that should be excluded. The content validity review resulted in most of the questionnaire prompts being ranked as highly relevant by the expert group; the CVI scale was measured as a 1.0 for mood and ease.

The factor analysis exploratory technique was used to investigate whether there were underlying variables influencing the patterns of subject responses. This analysis was completed using a sample size of 40 participants; the 20 items were examined and PCA was used for factor extraction. The investigators used Bartlett's test for equality of variance which produced a score of (p G .001). The Kaiser-Meyer-Olkin (KMO) test was also used and produced a score of 73.8; the KMO score indicated that the sampling was adequate. Ultimately, five of the factors were not used because they produced a 1.0 eigen value which is considered the lowest viable score; they accounted for 73.8% of variance (Table 3). The communalities varied between 0.58 and 0.72. The factor analysis results from this study were comparable to analogous studies.

**Table 2:** Internal Consistency (Reliability) coefficient of the child temperament scale.

Scale name	No. of Items	Questions in each item	Cronbach's Alpha
**Child Temperament tool**	20	All questions	0.834
**Factor 1**	5	Q1, Q2, Q3, Q4, Q5	0.781
**Factor 2**	6	Q6, Q7, Q8, Q9, Q10, Q11	0.462
**Factor 3**	3	Q12, Q13, Q14	0.586
**Factor 4**	2	Q15, Q16	0.859
**Factor 5**	4	Q17, Q18, Q19, Q20	0.772

**Table 3:** Exploratory factor structure of the child temperament questionnaire: Loadings for each factor and each item in the model with 5 factors after a varimax rotation and factor extraction using principle components analysis (N = 40).

Variable	Factors and factors loading				
	1	2	3	4	5
1. It is easy for me to calm or soothe your baby when he/she is upset?	0.907				
2. It is easy for me to predict when your baby will go to sleep and wake up?	0.820				
3. It is easy for me to predict when your baby will become hungry?	0.774				
4. It is easy for me to know what's bothering your baby when he/she cries or fusses?	0.598				
5. My baby get fussy and irritable – for short period	0.571				
6. My baby cry and fuss in general?		0.898			
7. My baby easily your infant get upset?		0.858			
8. My baby require, other than for caregiving (feeding, diaper changes, etc.)?		0.643			
9. My baby typically responds to a new person		0.566			
10. My baby typically responds to a new Place		0.549			
11. My baby adapt to things (e.g., bath, solid food, new person, new place) eventually		0.514			
12.My baby plays well by him/herself, when left alone			0.754		
13. My baby gets upset (e.g., before feeding, during diapering, etc.), vigorously or loudly			0.748		
14. My baby in trying to get my attention when I am are busy			0.539		
15. My baby is active in general				0.892	
16.My baby smile and make happy sounds?				0.882	
17. My baby respond to disruptions and changes in everyday routine, such as when you go to church or a meeting, on trips, etc.?					−0.711
18. My child's mood is changeable?					0.690
19. My child become good when people play or talk to him/her?					0.535
20. My baby difficult on the average.					−0.492
Percent of variability explained	39.9%	11.8%	9.4%	7.4%	5.3%

## 4 Internal consistency

The researcher describe this factorial analysis as a robust because Cronbach's alpha values were good, the first's alpha (0.86) is considered good and the Cronbach alpha for other factors al were (0.78,.77, 0.46 and 0.59). For more information (see Table 2). To summarize, the temperament instrument has an acceptable reliability for the whole instrument and the subscales**.**

## 5 Discussion

The translated ICQ was created through the process of translation-back translation, reviewed by the investigators, and tested using a small test sample. After review, the translation was considered to have the equivalent meaning in the Arabic language as in the original English-language version. It can be considered the standard for use among the Jordanian population. The aim of this research study was to examine the psychometric qualities of an Arabic language translation of the ICQ and present an analysis of the research study results using the translated ICQ in Jordan. While the translated version of the ICQ was validated and considered to have conceptual equivalence, there are still significant differences between the societies where the ICQ was tested, and it should be noted that parent-infant interaction, paternal-responsiveness to infant, and interpretation of infant temperament on a scale from easy to difficult, might vary widely according to environment as child rearing, normalities and abnormalities, can be understood quite differently across cultures [15].

Due to the small sample size, and the use of a convenience sample the results from this study are not generalizable. Additionally, the original study used a sample size of 523 (mother-children subjects) compared with 40 (mother-children subjects) from this study; this difference may have led to a significant difference in item level distribution by factor. However, the results from this study and the use of the ICQ are still important because it still produced valid data, though closer to the minimum number of subjects.

The research has shown that the infant temperament can be considered a contributing factor to successful or unsuccessful socialization and adaptation to the external environment throughout the lifetime making this measurement at an early stage very important. This relationship makes it worthwhile to investigate the parenting practices in various environments and consider improvements to the parent-infant relationship in culturally relevant ways and as a result, potentially improving the infant's life experience, externalizing behaviors, social development and lessening the extenuation of problematic behaviors [6].

### 5.1 Implications and recommendations for pediatrics

This research study described the translation of the ICQ into the Arabic language for use in the research and clinical setting. The ICQ is a useful tool for evaluating infant difficulty by describing and summarizing parents' ratings [9]. This measurement can then be used to determine potential interventions that health care practitioners might recommend improving the parent-infant reciprocal relationship. Improvement of parental practices, via mediations such as targeted responses to infant distress, could have long term benefits related to externalizing behavior in children, and could also help empower parent's who are at risk of withdrawal when faced with difficult infants, further risking infant development [6,7]. Interventions related to modification of child-rearing practices are also significant because the quality of caregiving has been linked to the formulation of the infant's view himself/herself in relation to the world, thus helping parent's mediate difficulties is a worthwhile endeavor [8]. The existing literature has also documented a relationship between infant difficulty level and later problematic behavior, making it a type of predictor, although not absolutely or singularly, for later disruptive behavior problems [16]. Due to the previously stated reasons, it is significant to measure and at the very least attempt to address infant temperament in a way that produces improved infant adjustment to various environmental factors and lessens distress; however, the impact of various actual intervention strategies would be the subject of another paper. What could be postulated is that the availability of the ICQ in various languages could help stakeholders provide more individualized methodologies to parents in need of techniques for successfully managing infant care.

### 5.2 Limitations

This study was limited with using very small size of the participants. Another limitation was using sample from homogenous population which may limit the generalizability of the results to sample from other countries.

## 6 Conclusion

The nursing field and its practitioners have a very important role with patients of all kinds. In nursing for infants, the parent-child, or caregiver-child relationship, is very significant and is often understood as bilateral, correlative, and interdependent. The measurement of infant temperament via self-report questionnaire can help provide important details about the parent-child or caregiver-child relationship including a providing a starting point for interventions that can change infant and parental responses. The research studies such as this study, can provide a starting point for changing that relationship for the better, producing an improved and more predictable relationship between the parent/caregiver and child.

## Declaration of competing interest

No conflict of interest.

## Registration of research studies

1. Name of the registry: Research Registry.
2. Unique Identifying number or registration ID: researchregistry6670.
3. Hyperlink to your specific registration (must be publicly accessible and will be checked): https://www.researchregistry.com/register-now#user-researchregistry/registerresearchdetails/60548e4ae6df0f001c9fadb8/
